# Antioxidant, Antimicrobial, and Other Biological Properties of Pompia Juice

**DOI:** 10.3390/molecules25143186

**Published:** 2020-07-13

**Authors:** Antonio Barberis, Monica Deiana, Ylenia Spissu, Emanuela Azara, Angela Fadda, Pier Andrea Serra, Guy D’hallewin, Marina Pisano, Gabriele Serreli, Germano Orrù, Alessandra Scano, Daniela Steri, Enrico Sanjust

**Affiliations:** 1 Institute of Sciences of Food Production, National Research Council, 07100 Sassari, Italy; yspissu82@gmail.com (Y.S.); angela.fadda@cnr.it (A.F.); paserra@uniss.it (P.A.S.); guy.dhallewin@cnr.it (G.D.); orru@unica.it (G.O.); 2 Department of Biomedical Sciences, University of Cagliari, 09100 Cagliari, Italy; mdeiana@unica.it (M.D.); gabrieleserreli@hotmail.it (G.S.); 3 Institute of Biomolecular Chemistry, National Research Council, 07100 Sassari, Italy; emanuelagigliola.azara@cnr.it (E.A.); marina.pisano@cnr.it (M.P.); 4 Department of Medical, Surgical and Experimental Medicine, University of Sassari, 07100 Sassari, Italy; 5 Department of Surgical Sciences, Molecular Biology Service, University of Cagliari, 09100 Cagliari, Italy; alessandrascano@libero.it; 6 PPD Pharmaceutical Industries, 37121 Verona, Italy; daniela.steri@googlemail.com

**Keywords:** Pompia juice, phenolics, chemical characterization, antioxidant activity, antimicrobial-antibiofilm activity, colon cancer cells

## Abstract

Pompia is a Citrus species belonging to Sardinian endemic biodiversity. Health benefits were attributed to its flavedo rind extracts and essential oils while the juice qualities have never been investigated. In this paper, the antioxidant, antimicrobial, and other biological properties of Pompia juice were studied. A combined LCMS/electrochemical/biological approach was used to clarify a still debated phylogeny of this species and to explain the role of its juice phenolic compounds. A closer phylogenetic relationship with lemon and citron, rather than oranges was suggested. Sensors-based electrochemical measures, together with LCMS qualitative and quantitative analyses, revealed a high contribution of ascorbic acid and phenolics with low redox potential, isorhamnetin 3-*O*-rutinoside, diosmin, and diosmetin 6,8-diglucoside, to antioxidant capacity. The biological assays demonstrated a marked effect of low concentration of Pompia juice against reactive oxygen species (ROS) starting from 50 µg mL^−1^, and a moderate capacity to reduce ROS damages on cell membrane. Treatments with Pompia juice also resulted in a significant reduction (20%) of the metabolic activity of SW48 colon cancer cells. Lastly, MIC, MBC, and MBIC antimicrobial assays demonstrated that Pompia and lemon juices have inhibitory and antibiofilm effects against the pathogenic bacteria *Pseudomonas aeruginosa*, *Streptococcus aureus*, and *Enterococcus faecalis*.

## 1. Introduction

*Citrus* species are extensively cultivated all over the world because of their high organoleptic and nutritional value. They are eaten as fresh or consumed as jams, candies, juices, and ingredients for sweets and desserts. Being rich in vitamin C and phenolics, especially flavonoids [1], the attention of the literature to citrus fruits antioxidant properties has considerably increased because of the evidence of a correlation between the intake of citrus fruits and a reduced risk to develop degenerative diseases, neurological and cardiovascular disorders, obesity, diabetes, and certain types of cancer [2,3,4,5].

The antioxidant activity has been widely studied in *Citrus* species and hundreds of papers showed that ascorbic acid (AA) and polyphenols are primarily responsible for this [6]. The combined electrochemical and mass-spectrometry approach suggested a preeminent role of vitamin C as antioxidant and a much more complex function of flavonoids and anthocyanins [7]. Phenolics from citrus fruits own important qualities, with a large number of beneficial effects on human health. Bergamot juice acts as anti-inflammatory agent in lipopolysaccharide-treated murine macrophages because of its high content of flavonoids [8], and blocks the proinflammatory actions induced by interferon-gamma and histamine on human keratinocytes [9]. Hydro-alcoholic extracts of *C. aurantium* peel significantly inhibited histamine- and dextran-induced edema in rats in a concentration-dependent manner, providing evidence of a possible therapeutic use [10]. *C. limon* therapeutic action has been studied by in vitro assays of ethanol extracts against a panel of microbes implicated in skin diseases [11], while the juices of *C. hystrix* and *C. maxima* were investigated for their anti-diabetic, cholinesterase, and tyrosinase inhibitory potential [12]. Studies on anti-tumor effects of citrus juice bioactive compounds were also performed: a chemo-preventive activity on neuroblastoma and hepatocellular carcinoma was attributed to phenolics of bergamot juice [13,14,15] and to flavonoids of Mexican lime juice on pancreatic cancer [16].

Among *Citrus* species, *Citrus limon* var. *pompia* Camarda var. nova [17], previously classified as *Citrus x monstruosa* [18], is a particular citron-like tree locally known as “Pompia.” It is an endemic species cultivated in Sardinia, primarily along the north-eastern coast of the Italian island. The tree has an ancient origin [19,20] and its cultivation has recently known a revival because of the use of fruit rind in Sardinian traditional sweets, classified as Slow Food [21]. For centuries, the local tradition has attributed to Pompia flavedo and its essential oils beneficial properties on health, even though no scientific evidence was available. Only recently, a chemical characterization of Pompia rind compounds has been carried out in the view of their incorporation in vesicular systems for skin delivery [22] and for the protection of oral cavity [23], while Pompia leaf essential oils were characterized to investigate the antimicrobial activity against food-related microorganisms [24]. Other peculiarities of this fruit are the characteristic flavor, which somewhat resembles that of lemon, the very thick albedo and the low quantity of juice, which is characterized by high acidity and low sugar content [18].

As far as we know, no information is available on the properties of Pompia juice. Therefore, the aim of this work is to investigate its putative antioxidant and antimicrobial capacity, and antiproliferative activity on colon cancer cells, in comparison with the well-known Lisbon lemon cultivar [25] and some previously studied orange varieties [7]. Once achieved a phenolics characterization, the antioxidant capacity of Pompia juice was measured, and electrochemical analyses, based on a previously developed sensor/biosensor system, were used to distinguish the AA and phenolics contribution to antioxidant activity [7,26]. The protective effect against reactive oxygen species (ROS) and enterocyte membrane damage was investigated using differentiated CaCo-2 intestinal cancer cells. Furthermore, with the evidence of the above mentioned antitumoral effects of others *Citrus* spp., the capacity of low concentration of Pompia juice to affect the metabolic activity of parental undifferentiated CaCo-2 cells and of SW48, both colon cancer cell lines, was studied. Lastly, we investigated the antimicrobial and antibiofilm activity of Pompia juice and the other citrus fruits against Gram-negative and Gram-positive bacterial strains, in comparison with specific antibiotic drugs.

## 2. Results and Discussion

Pompia juice has such organoleptic characteristics that it has traditionally always been considered a waste product of the fruit. The yield is very low (12.3 ± 2%), the pH is 2.3 ± 0.5, the titratable acidity is 6.9 ± 0.2% citric acid equivalents, and TSS are 7.2 ± 0.3° Brix. The taste is particularly sour and bitter.

### 2.1. Chemical Characterization of Citrus Juices

The chromatograms of phenolics detected in Pompia, lemon, and orange juices are reported in Figure S1, while the quantification of the most represented compounds (all the phenolics over the LOQ of LCMS system) is in Table 1.

The chromatograms of phenolics detected in Pompia, lemon, and orange juices are reported in Figure S1, together with the complete list of 46 (21 in Pompia, 20 in Lemon, 14 in Hamlin, 14 in Sanguinello and 16 Moro) identified molecules, together with their retention time (RT) (Table S1). Because of the more precise analytical method adopted, this characterization represents a significant upgrade compared to the previous one for the same orange varieties [7]. The higher peak in the Pompia juice (RT = 11.15 min) is a quinic acid derivative but, since a better characterization of this molecule was not possible, its quantification could not be provided. According to Table 1 and Table S1, the phenolic composition of Pompia juice is more similar to lemon than to the oranges. The most represented flavonoid in the Pompia juice, not detected in the other juices, is the chrysoeriol-6,8-di-*C*-glucoside also known as stellarin-2 (109.2 mg L^−1^), described for the first time in the juice of the lemon *cv* Verna [27]. Isorhamnetin-7-rutinoside, diosmetin-6,8-dihexoside, and diosmin (79.4, 54.5, and 52.6 mg L^−1^ respectively) are also highly represented in Pompia, significantly less in lemon, and are absent in the oranges. Rhoifolin-4-glucoside was also found only in the juice of Pompia, while 31.4 mg L^−1^ of rhoifolin, the corresponding aglycone, was detected in the lemon juice. Hesperidin, generally responsible for citrus juices cloudiness [28], is the only molecule common to all the juices, found in a very low concentration in Pompia but highly represented in all orange varieties (422.8, 366.4, and 182.3 mg L^−1^ in Hamlin, Sanguinello, and Moro respectively). No anthocyanins were found in the Pompia juice. As previously said, Pompia has been considered a citrus variety belonging to *C. limon* species [17], but there are not definitive conclusions on this taxonomy and the most recent studies, based on karyological [20] and genetic [19] analyses suggested that Pompia is a cross between sour orange and citron. The provided phenolics characterization of Pompia juice adds elements but cannot solve this puzzle since many molecules identified in Pompia were also found in lemon juice, while some are typical of citron (*Citrus medica* L.) [29,30], and some others characterize bergamot [13,28], another species of still uncertain botanical origin, defined as a hybrid between sour orange and lemon [15]. On the other hand, similarities can be found between Pompia [24] and bergamot [31] essential oil composition.

### 2.2. Antioxidant Capacity and AA Content Determination in Citrus Juices

Table 2 shows the antioxidant capacity (Table 2A) of different juices, obtained with the SB, DPPH, and ABTS methods. It also provides data concerning ascorbic acid content (Table 2B) measured by SB, titrimetric, and HPLC methods.

According to SB, Pompia and lemon showed similar antioxidant capacity (4.98 and 4.59 mmoL equivalents of AA L^−1^ of juice respectively), higher than Hamlin, the blond orange variety, Sanguinello and Moro (Table 2A). Similar trends were obtained with the three methods of analysis, even though DPPH, and especially ABTS, showed values statistically higher than SB for the same samples. This difference was expected since the SB system oxidized only the molecules with a redox potential ≤ + 500 mV, highlighting the role of AA and phenolics with low redox potential, those which act first as radical scavengers. Differently, DPPH and ABTS methods consider a wider pool of compounds, consistent with a reduction potential of +680 mV [32]. To better understand the relationship between total phenolic content and the phenolic contribution to the antioxidant activity, we correlated first the total phenols (determined with LCMS from Table 1) with the total antioxidant activity (measured by the three systems, from Table 2), and then the total phenolics with the phenolic contribution measured by the sensor (from Table 2). In all the cases we did not find any significant correlation, as in many other papers in the literature.

The difference in antioxidant capacity among species cannot be simply attributed to a different content of total phenolics, but should be ascribed to AA content and to the quality of phenolic compounds. Only the redox potential is indication of their reducing power [33] so, what matters is the class of molecules to which they belong and their relative quantity in the juice. The AA oxidation occurs at a very low potential, close to zero mV [7,26,34]. The AA content in Pompia is higher than in lemon and oranges, so its contribution to antioxidant capacity is decisive (Table 2B).

**Figure 1:**
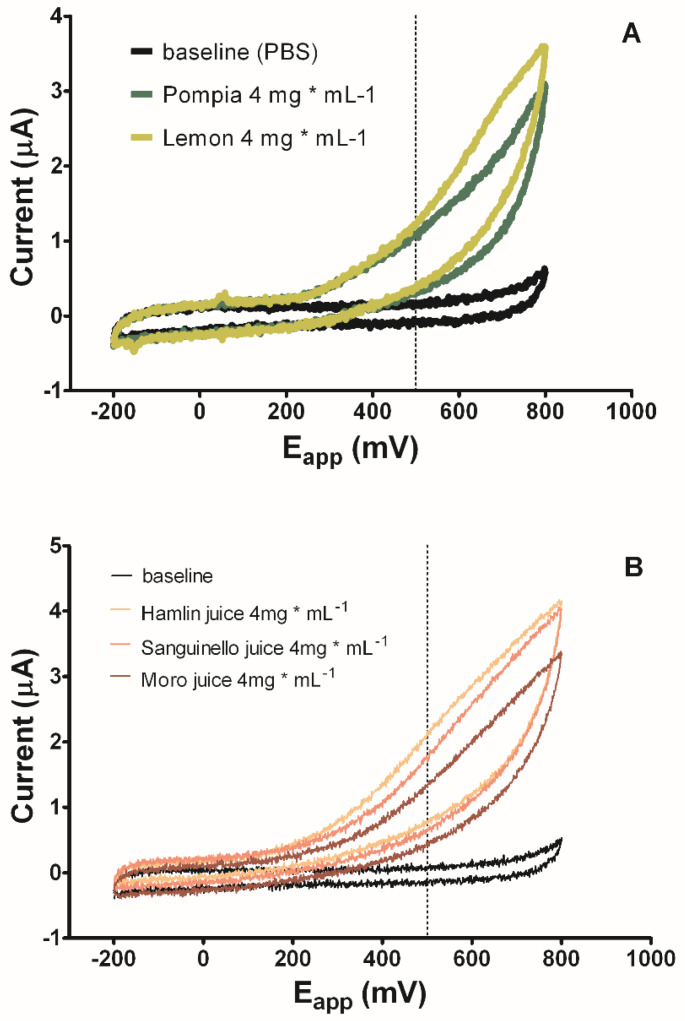
Cyclic voltammetry, with a scanned potential range (E_app_) comprised between –200 mV and +800 mV vs. carbon pseudoreference, in the absence (PBS black line) and in the presence of 4 mg mL^−1^ of Pompia (green line) and lemon (yellow line) juices (**A**), and in the absence (PBS black line) and in the presence of 4 mg mL^−1^ of Hamlin (yellow line), Sanguinello (orange line), and Moro (brown line) juices (**B**).

In Figure 1, the cyclic voltammetric patterns recorded for Pompia and lemon (A), and for oranges (B), revealed that the oxidation in juices started from +80 to +150 mV, a value mainly consistent with the AA redox potential [26]. The voltammograms had similar shapes but the currents, recorded at +500 mV, by different juices, varied from 1 to 2 µA. These differences should be attributed to the activity of phenolics with different redox potential. According to CVs of some of the most represented compounds, the antioxidant activity of Pompia is principally due to isorhamnetin 3-*O*-rutinoside (oxidation starts at +80 mV), diosmin and diosmetin 6,8-diglucoside (oxidation starts at +400 mV and +460 mV respectively) (Figures S2, S3, and S4). Unfortunately, the standard of one of the most represented compounds in the Pompia juice, the Stellarin-2, was not available, so we can only rely on the literature to speculate on its antioxidant capacity. This *C*-glucoside, previously identified in other citrus species [27,28,35] was found in high concentration in Pompia juice. Based on these studies, Stellarin-2, as well as similar flavonoid derivatives, contributes to the reducing ability bearing a double bond at 2.3 position of the *C-*ring. Unfortunately, in the absence of a standard, we have not been able to determine the redox potential of this molecule nor, consequently, its specific contribution to antioxidant activity.

Lemon juice owes its antioxidant capacity mainly to AA, isorhamnetin 3-*O*-rutinoside, and hesperidin (oxidation starts at +370 mV). Orange juices have deeply different phenolics composition in comparison to Pompia and lemon, and their antioxidant capacity was flesh color dependent: 95% of flavonoids in Hamlin, Sanguinello, and Moro is represented by hesperidin and narirutin which relatively contribute to antioxidant capacity being oxidized starting from +370 mV and +520 mV, with oxidation peaks at +680 mV and +820 mV, respectively [7,26]. The polyphenols of Sanguinello and Moro contribute 15% and 26%, respectively, to antioxidant capacity, although the phenolics content of Sanguinello is greater than Moro. Moro contains a high level of anthocyanins (especially cyanidins whose oxidation begins at about +280 mV and have redox peaks at about +400 mV [26], which oxidize to a lower potential than Sanguinello flavonoids, hesperidin, narirutin (>600 mV), and naringin (+520 mV). It has already been shown that the most of organic acids, like citric acid [34] or quinic acid (Figure S5) did not give any contribution to antioxidant capacity.

### 2.3. Protective Effect of Pompia and Other Citrus Juices Against ROS and Enterocytes Membrane Damage

**Figure 2:**
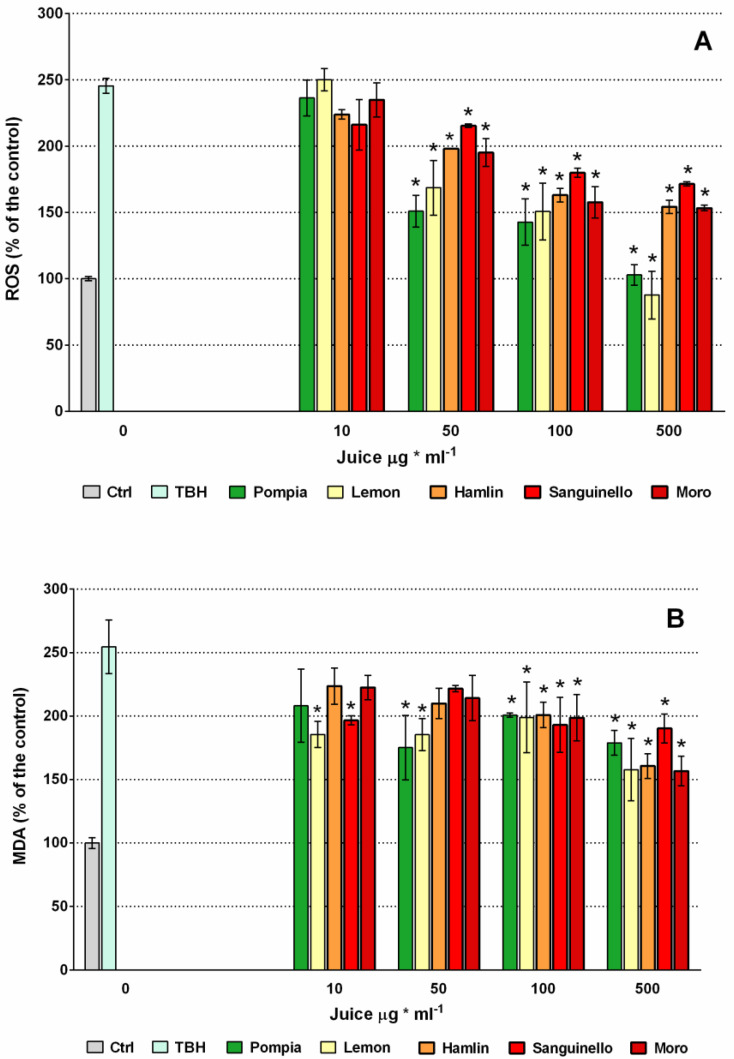
Evaluation of 2.5 mM TBH oxidative stress on differentiated CaCo-2 cell monolayer by determination of intracellular reactive oxygen species (ROS) production (**A**) and malonyldialdehyde (MDA) level (**B**), in the absence and in the presence of Pompia and other juices. * *p* ≤ 0.01 vs. TBH.

The ability of the juices to exert an antioxidant action was further tested in a more biologically relevant experimental system as the cell cultures. Differentiated Caco-2 cells were chosen as model of human enterocytes. Control experiments were first carried out to assess a potential juice cytotoxicity, but cell viability remained unchanged in the presence of all the tested juices, compared to untreated cells (100% of viability), in the concentration range of 10–500 µg mL^−1^ (Figure S6). Then, the potential protective effect of the juices against TBH-induced oxidative damage in CaCo-2 cell monolayers was investigated (Figure 2A). A highly significant steep increase of ROS production was caused by 2.5 mM TBH, in comparison with the untreated cells (control), starting from 15 min of incubation. TBH originates ROS in the reaction mixture that catalyze the peroxidation of membrane lipids [36]. In the presence of the juices, ROS production significantly decreased starting from 50 µg mL^−1^ in comparison with positive control (TBH 2.5 mM alone), and gradually lowered as the juice concentrations grew; at 500 µg mL^−1^, Pompia and Lemon were able to bring the ROS production back to untreated cells value. Orange juices appeared much less effective. The ability of the citrus juices to protect CaCo-2 cell monolayers against TBH oxidative injury was also assessed by measuring malonyldialdehyde (MDA) level after 2 h of incubation with 2.5 mM TBH, when an advanced peroxidation process had damaged the membrane lipid fraction giving rise to detectable oxidation products as MDA [37]. A 2.5-fold increase of MDA level compared with untreated cells (control) was observed in the culture medium of TBH-treated cells (Figure 2B). Treatment with the juices significantly inhibited MDA production starting from 10 µg mL^−1^ in the case of Sanguinello and lemon and from 50 µg mL^−1^ for all the juices.

These data indicate that Pompia juice is highly effective against ROS production and damaging action, in accordance with the capacity of Pompia rind extracts to prevent oxidative damage, promoting the viability of human keratinocytes and mouse fibroblasts [22]. The antioxidant activity of the Pompia juice in the cell cultures may be related to its radical scavenging ability, as indicated by the sensor, DPPH and ABTS assays. It is noteworthy that Pompia juice, even showing the best scavenging ability in the chemical assays, did not protect the cell membrane more than the other juices. These results further support the opinion that the action of an antioxidant is greatly dependent on the reaction environment and it may be exerted through more than one mechanism in a complex environment such as a biological system.

### 2.4. Influence of Pompia and Lemon Juices on Metabolic Activity of Colon Cancer Cells

In our study, CVS was used to evaluate CaCo2 and SW48 cancer cells number when exposed to a preventive treatment with increasing concentration of citrus juices (Figure S7), as this non-enzymatic assay allows the estimation of the number of viable cells, adherent and colonies, since the amount of dye absorbed depends on the total DNA content [38]. Differently, the MTT assay may result in either underestimation or overestimation of the fraction of live cells with the result depending on the type of cells [39].

**Figure 3:**
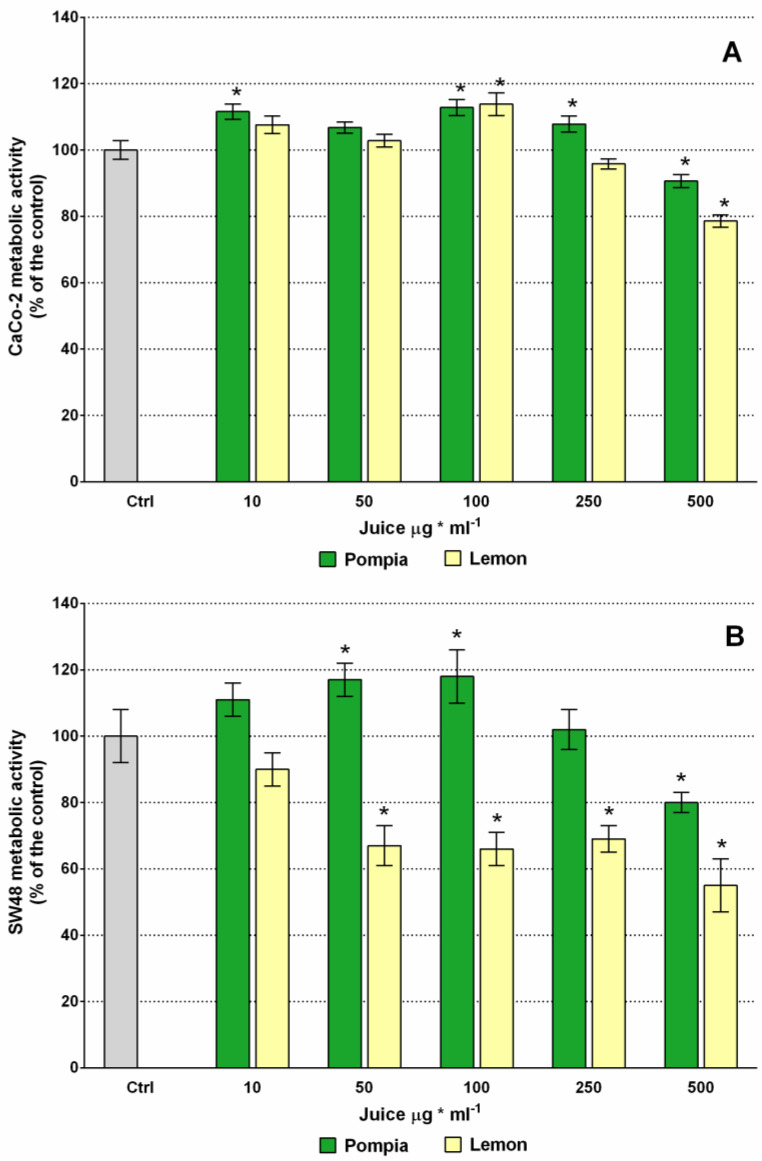
Effect of Pompia and lemon juices on metabolic activity of parental undifferentiated CaCo-2 (**A**) and SW48 (**B**) colon cancer cell lines. * = p ≤ 0.01 *vs* control.

MTT assay was used instead to assess the metabolic activity linked to mitochondria functional state [39,40]. Orange juices of all varieties being totally ineffective on both cell lines, only the results of Pompia and lemon were reported on the graphs. The exposure of CaCo-2 to Pompia and lemon juices did not reduce the number of cancer cells, but a 15–40% and 10–20% increase was actually observed for Pompia and lemon juice respectively (Figure S7A). Differently, CaCo-2 metabolic activity (Figure 3) did not appear significantly changed by juice treatments, with the exception of a reduction of less than 10% and of 20% at 500 μg mL^−1^, for Pompia and lemon respectively (Figure 3A).

No significant difference against control was observed in the SW48 cells number (Figure S7B). Otherwise, the SW48 metabolic activity progressively increased when they were exposed to 10–50 and 100 μg mL^−1^ of Pompia juice, then it rapidly decreased reaching the control values at 250 μg mL^−1^ and then it was reduced by 20% when the cells were treated with 500 μg mL^−1^ (Figure 3B). Lemon juice has proven to be much more aggressive toward SW48 cells: 50 μg mL^−1^ reduced it by 30%, up to a maximum of 50% at 500 μg mL^−1^.

The effects of natural compounds on cancer cells viability can vary depending on cell types, and the mechanisms involved in cellular responses are still largely unknown. In this paper, dose-dependent antioxidant and anti-proliferative effects were observed, in accordance with previous studies with other natural compounds. As already reported [41,42,43] the consumption of citrus juice and their phytochemicals plays an important role in the prevention and treatment of various cancers, including colon cancer. Moreover, their greater or lesser effect related to the tumor staging should not surprise [41]. Our treatments with low doses of Pompia and lemon had different effects on both colon cancer cell lines, showing a greater effect on SW48, cells with metastatic capacity. Other authors obtained similar data with bergamot juice but with ten times higher doses [13,44]. Moreover, the treatment with low concentration of Pompia juice (≤100 µg mL^−1^) increased the number of CaCo2 cells and the metabolic activity of CaCo2 and SW48. Even though it was similarly reported [44], we do not have any definite explanation for this effect, which would deserve extra investigations. Further studies on neuroblastoma cells showed that bergamot juice significantly affected SK-N-SH and Lan-1 cell proliferation in vitro starting from 48 h of treatment [13]. Studies on human hepatocellular carcinoma showed that the growth rate of HepG2 cells is reduced by high concentrations of bergamot juice by the activation of apoptotic pathways [14]. The induction of apoptosis of Panc-28 cells, demonstrated by a high ratio Bax/Bcl-2, is the mechanism underlying the proliferation inhibitory capacity of Mexican lime against pancreatic cancer [16].

### 2.5. Antimicrobial Assays

The result of Kirby-Bauer diffusion procedure preliminary test is reported in Table 3. It indicates that orange juices were ineffective (data not shown) and that Pompia and lemon juices showed similar antibacterial activity: they inhibited Gram-positive strains *Streptococcus aureus* DSM1104 and *E. faecalis DSM 2570* and, the most important, they exerted a strong inhibiting action on the nosocomial infection-associated pathogen, *P. aeruginosa* (Gram-negative). *E. coli and K pneumoniae* resulted not sensitive to citrus juices.

#### MIC, MBC, and Antibiofilm Activity (MBIC) Evaluation of Citrus Juices

According to Kirby-Bauer method, only the strains sensitive to citrus juices were used for MIC and MBC tests. As reported in Table 4, these juices caused bacterial growth inhibition (MIC) in a concentration range from 2 to 4 mg mL^−1^: *S. aureus* represented the most citrus-sensitive strain. For all tested bacteria, both juices were unable to reveal a bactericidal effect inside the tried concentrations range (MBC > 4 µg µL^−1^).

Table 4 also reports the minimum biofilm inhibition concentration between Pompia and lemon juices. A significant difference between the two juices on the ability to interfere in the biofilm formation was observed. As shown in Table 4, Pompia juice reported lower MBIC values (1 log_2_) than lemon.

These results demonstrated that Pompia and lemon juices have an inhibitory effect against some pathogenic bacteria. Previous studies had already shown that Pompia rind extracts and lemon juice inhibited bacterial proliferation [11,23,24,45]. Although in this work Pompia and lemon juices did not show any bactericide activity, a bacterial growth inhibition and an antibiofilm effect have been observed. In this context, the antibiofilm propriety could be interesting since these microbial species are frequently associated with “biofilm-related diseases” in humans [46,47,48]; in in vitro experiments, as well as in the clinical practice, the microbial sessile status is often more resistant to conventional biocides and the host immune system, than their planktonic form [49]. For this reason, these infections are difficult to treat and could considerably rise the healthcare costs, as reported by several studies [50] and a “natural drug” able to inhibit the biofilm formation is highly sought.

## 3. Materials and Methods

### 3.1. Fruit Samples and Juice Preparation

Pompia (*Citrus limon* var. *pompia* Camarda var. nova), lemon (*Citrus limon* (L.) Burm. f.) cultivar Lisbon, and orange (*Citrus sinensis* (L.) Osbek) cultivars Hamlin, Sanguinello, and Moro, were hand-harvested from December 2018 to February 2019, when commercially mature, in the experimental orchard of the Institute of Sciences of Food Production, in Oristano (Latitude: 39°53′58.45″ N; Longitude: 8°35′35.74″ E; 8 m a.s.l.) Sardinia, in Italy. Fruit was delivered to the laboratories immediately after harvest. There, medium size fruits, free from rind defects were randomly selected, placed in plastic trays, and left overnight at 17 °C. The juice prepared by squeezing fresh fruits, was filtered with a kitchen strainer, immediately stored in ultra-freezer at −80 °C, and then lyophilized. Freeze dried samples were fully rehydrated immediately before chemical analyses, phenolics detection, and quantification, in vitro calibrations and antioxidant activity determination. For biological tests, dried juices were dissolved in PBS and then diluted with the specific medium.

### 3.2. Chemicals and Cell Lines

All the reagents and solvents were of reagent grade, used without any further purification. Ascorbic acid (AA), ascorbate oxidase (AOx, EC 1.10.3.3), 2,2-diphenyl-1-picrylhydrazyl (DPPH) free radical, Rutin, 2,2’-Azino-*bis*(3-ethylbenzothiazoline-6-sulfonic acid, diammonium salt (ABTS), 2′,7′- dichlorodihydrofluorescein diacetate (H_2_DCFDA), *tert*-butyl hydroperoxide (TBH), trichloroacetic acid (TCA), 2-thiobarbituric acid (TBA), methanol, 1,1,3,3-tetraethoxypropane (TEP), thiazolyl blue tetrazolium bromide (MTT), isopropanol, streptomycin, penicillin, ampicillin, oxacillin, cloxacillin, rifampicin, oxytetracycline, and L-glutamine were purchased from Sigma-Aldrich (Milan, Italy). The phosphate-buffered saline (PBS) solution was made using NaCl (137 mM), NaOH (2.7 mM), Na_2_HPO_4_ (8.1 mM), and KH_2_PO_4_ (1.47 mM) from Sigma and then adjusted to pH 7.4. Dulbecco’s modified Eagle’s medium (DMEM), RPMI 1640 and fetal bovine serum (FBS) were purchased from Euroclone S.p.A. (Pero, Milan, Italy).

CaCo-2 cells were obtained from the European Collection of Cell Cultures (ECACC, Salisbury, UK). SW48 were purchased from the American Type Culture Collection (ATCC, LGC Standards S.r.l., Milan, Italy).

### 3.3. Chemical Analyses

pH was measured by a pH-meter (Orion, 420A, Champaign, IL, USA). Titratable acidity (TA) was determined by Mettler Toledo™ DL15 Potentiometric Titrator (Milano, Italy) and expressed as percentage of citric acid equivalents. Total soluble solids (TSS) were determined by a digital refractometer Atago PR-101 (Atago, Tokyo, Japan) at 20 °C and expressed as Brix.

### 3.4. Phenolic Characterization of Citrus Juices

Separations of phenolic compounds were performed using a 1200 series HPLC liquid chromatographic system (Agilent Technologies, Milano, Italy). A Phenomenex Kinetex C18 column (100 mm × 2.1 mm, 2.6 µm, 100 Å, Milano, Italy) was used for the chromatographic separation. The flow rate was 0.250 mL/min during a 60-min period with an injection volume of 5 µl. Mobile phases were: 1 mM ammonium acetate with 0.1% acetic acid (A) and methanol with 0.1% acetic acid (B). A linear gradient elution of solvents was applied with the following program: 0 min, 85% B; 20 min, 75% B, 40 min 50%B, 50 min 30% B. The column was equilibrated for 8 min prior to each analysis. The chromatographic system was coupled to an OrbiTrap high resolution mass spectrometer (Waltham, MA, USA) equipped with heated electrospray ionization probe HESI-II (Thermo Scientific, Bremen, Germany) operating in both positive and negative ion mode. Parameters of the ion source were as follows: spray voltage, 3.0 kV (positive ionization), 2.5 kV (negative ionization); sheath gas flow rate (N_2_) 35 units; capillary temperature 300 °C; S-lens RF level 50; heater temperature 350 °C. Full MS acquisition was performed with resolution power 70000 FWHM with mass accuracy of 5 ppm. The MS parameters were: AGC target 3e^6^, maximum injection time (IT) 200 ms, and scan range 100–1500 *m*/*z*. The Xcalibur™ software (software version 4.3, Thermo Scientific, Bremen, Germany) was used to control the instruments and to process the data. Mass deviation were calculated as parts per million [(calculated mass-experimental mass/calculated mass)] and were found to be below 5 ppm.

Peaks of the phenolic compounds were characterized on the basis of their retention time (*t*_R_) values, UV/Vis spectra, and HRMS spectra. Individual phenolic compounds were quantified using calibration curves of the respective reference compounds detected by DAD (Agilent, Santa Clara, CA, USA) at 270 and 320 nm.

### 3.5. Analytical and Biological Determination of Antioxidant Properties of Pompia and Other Citrus Juices

The antioxidant properties of Pompia and other citrus juices were investigated by both analytical and biological tests.

#### 3.5.1. Analytical Detection of Antioxidant Activity and AA Content

The antioxidant capacity was evaluated by both a non-spectrophotometric and a spectrophotometric technique, since methods based on color changes of chromogen radicals could be influenced by the presence of red pigments (anthocyanins) in the juice of blood oranges (Moro and Sanguinello). The capacity of the tested samples to scavenge the DPPH radical was determined by electron paramagnetic spectroscopy (EPR) according to [51] with some modifications, and results were expressed as mmoles equivalents of AA L^−1^ of juice on the basis of a calibration curve (r^2^ = 0.99); second, the ABTS radical cation decolorization assay was performed according to [52] and the antiradical activity was expressed as mmoles equivalents of AA L^−1^ of juice. AA was detected by titrimetric and chromatographic methods and expressed as mg L^−1^ of juice [7].

#### 3.5.2. Electrochemical Determination of the Contribution of AA and Phenolics to Antioxidant Capacity

Amperometry and cyclic voltammetry (CV) were used to determine the contribution of AA and phenolics to antioxidant activity. A previously developed fullerene C_60_-nanostructured sensor/biosensor system (SB), based on ascorbate oxidase (AOx) enzyme [26,53], was used to simultaneously determine the antioxidant activity and the AA content of all fruit juices, by distinguishing between AA and phenolics contribution to antioxidant capacity. An exhaustive description of the SB and its working principle is reported in [7] and [26]. All electrochemical measures were performed using a four-channel system (eDAQ Quadstat, e-Corder 410, and Echem software, eDAQ Europe, Warszawa, Poland) placing the SB in a cell, a glass beaker containing 20 mL of air-bubbled PBS at pH 5.6 which is the optimal pH for the AOx activity. The SB system operated at a constant applied potential (E_app_) of +500 mV (vs. carbon pseudo-reference electrode), a value that, according to [54], enables to determine the antioxidant capacity of phenolics, sugars, organic acids and, in general, of any molecule that can be oxidized at this specific potential. All the molecules with a redox potential higher than +500 mV were not accounted for. The quantification of AA and antioxidant capacity was obtained by injecting 100 μL of citrus juice into a 20 mL PBS solution. The recorded currents were plotted on an AA calibration curve, with concentrations ranging between 0 and 100 μM, and converted to express the values of antioxidant capacity as mmoles equivalents of AA L^−1^ of juice and AA content as mg L^−1^ of juice.

Moreover, CVs were carried out in order to investigate the electrochemical behavior of fruit juices, at the WE surface vs. the Ag/AgCl Ref, at a scan rate of 100 mV. CVs were executed from −200 mV to +800 mV. CVs of the most represented phenolics were carried out from −1 V to +1 V.

#### 3.5.3. Evaluation of Protective Effect of Pompia and Other Citrus Juices Against ROS and the Enterocytes Membrane Damage

Biological assays were performed to study the protective effect of citrus juices against ROS and the enterocytes membrane damage. Caco-2 cells, at passage 45–60, were plated at a density of about 1 × 10^5^ mL^−1^ and used 21 days post seeding. Once differentiated, Caco-2 cells acquire morphological and functional characteristics of mature enterocytes [55]. Differentiated CaCo-2 cells grew in Dulbecco’s modified Eagle’s medium (DMEM), supplemented with 2.5% of heat-inactivated fetal bovine serum (FBS), 100 U mL^−1^ penicillin and 100 μg mL^−1^ streptomycin, at 37 °C under a humidified atmosphere of 95% air and 5% CO_2_.

##### Evaluation of Potential Toxic Activity of Citrus Juices

An MTT assay was performed using differentiated Caco-2 cells [56], in order to evaluate any toxic activity of tested juices. Cells were seeded in 96-well plates (5 × 10^4^ viable cells mL^−1^ in 100 μL), exposed to various concentrations of juices between 10 and 500 µg mL^−1^ in medium, and incubated for 72 h. After incubation, the medium was removed and 100 μL of MTT solution (5 mg mL^−1^ of fresh medium) was added and left at 37 °C for 6 h. The medium was then aspirated, 100 μL of dimethyl sulfoxide (DMSO) was added in each well, and the absorbance was read at 570 nm by a Tecan micro plate reader (Infinite 200, Salzburg, Austria). Percentage of cell growth was calculated by normalizing the absorbance of treated cells to corresponding control.

##### Determination of Intracellular ROS Production and MDA Level

Intracellular ROS production was monitored in differentiated CaCo-2 cells seeded in 96-well plates [37]. The old culture medium was removed, cells were washed with 200 µL of PBS and incubated for 30 min in PBS with 10 µM of H_2_DCFDA. H_2_DCFDA was then removed, the cells were treated with juices at concentration ranging from 10 to 500 µg mL^−1^ and, 30 min later, with a 2.5 mM TBH aqueous solution. ROS production was monitored by reading the fluorescence emitted after 120 min of incubation, using a Tecan micro plate reader at a controlled temperature of 37 °C. The reading was performed using an excitation of 490 nm and an emission of 520 nm. After 120 min the supernatant was collected and malonyldialdehyde (MDA) level was measured with the TBARS test with HPLC quantification, as previously described [57]. Briefly, 100 µL of 10% TCA was added to 400 µL of PBS supernatant in a microcentrifuge tube; samples were mixed and left at room temperature. After 20 min, 200 µL of 0.6% TBA was added; the samples were incubated at 90 °C for 45 min and then centrifuged at 5000 *g* for 15 min at 4 °C. Aliquots of the supernatant were injected into an Agilent 1100 HPLC system equipped with a diode-array detector (HPLC-DAD) and analysis was carried out using a Varian (Middelburg, The Netherlands) column, Inertsil 5 ODS-2, 150 × 4.6 mm; the mobile phase was a mixture of KH_2_PO_4_ 50 mM pH 7/MeOH (65/35, *v*/*v*) at a flow rate of 1 mL min^−1^. The adduct MDA-TBA was revealed at 532 nm. A standard curve was prepared with the samples using a TEP solution in PBS (0.05–10 µM; r^2^ = 0.99).

### 3.6. Evaluation of Citrus Juices Effect on Cancer Cells Metabolic Activity

Undifferentiated CaCo-2 and SW48 colon cancer cells were used to study the effect of citrus juices on cancer cells metabolic activity. Undifferentiated CaCo2 were grown in DMEM, containing 10% FBS, 1% penicillin (100 U mL^−1^)/streptomycin (100 μg mL^−1^), and 1% L-glutamine at 37 °C under humidified 5% CO_2_/air. For experimental studies, cells at passage 25–30 were plated at a density of about 5 × 10^4^ mL^−1^ and used 24 h post seeding.

SW48 cell lines were grown in RPMI 1640 medium, containing 10% FBS, 1% penicillin (100 U mL^−1^)/streptomycin (100 μg mL^−1^), and 1% L-glutamine at 37 °C under humidified 5% CO_2_/air. For experimental studies, cells at passage 7–10 were plated at a density of about 1 × 10^5^ mL^−1^ and used 24 h post seeding.

Cells were first exposed to increasing concentrations of juice (10–500 μg mL^−1^) for 24 h. CaCo_2_ and SW48 cell number was evaluated using the crystal violet staining assay (CVS) as described by [58]: cells were fixed for 20 min at room temperature with 4% paraformaldehyde (PFA), stained with 0.1% crystal violet in 20% methanol for 20 min, washed with PBS, solubilized with 10% acetic acid, and read at 595 nm in a Beauty Diagnostic microplate reader.

The MTT assay was used to assess the metabolic activity as previously reported [40]: cells were incubated with 100 µL (0.05 mg mL^−1^) of MTT, and the cultures were allowed to incubate at 37 °C for 3 h. The MTT was removed and the formazan crystals were dissolved in 100 µL of isopropanol. The color was read at 570 nm using a microplate reader (Sunrise™ Absorbance Reader-TECAN).

Percentage of cell growth and metabolic activity were calculated by normalizing the absorbance of the treated cells to corresponding control.

### 3.7. Antimicrobial Activity

The antimicrobial activity of citrus juices was evaluated as already reported [59]. All bacterial strains *Escherichia coli* DSM 1103, *Pseudomonas aeruginosa* DSM 1117, *Klebsiella pneumoniae* DSM 681, *Streptococcus aureus* DSM 1104, *and Enterococcus faecalis* DSM 2570 were purchased from DSMZ (German Collection of Microorganism and Cell Cultures, Braunschweig, Germany). Fifty microliters of the frozen bacterial suspension was inoculated in Mueller Hinton and Schaedler Broth (Microbiol, Uta, Cagliari, Italy) and incubated at 37 °C until the growth middle-logarithmic phase; *E. faecalis* was inoculated in Schaedler Broth and incubated at 37 °C with 5% CO_2_.

A preliminary evaluation of all juices was performed by Kirby-Bauer disk-diffusion modified method [60]. Briefly, 15 mL of agarized Muller Hinton or Schaedler medium (Microbiol) at 55 °C were added to a 90 Ø mm Petri dish. Prior to agar solidification, four sterile iron rivets (Ø 10 mm diameter and 2 mm thick) were put into the agar mixture so that, once removed from the solidified agar, they leave 100 µL wells. A 5 × 10^7^ CFU mL^−1^ standardized inoculum of each strain was inoculated onto the plate surface using a sterile swab, then 100 µL of a 4 mg L^−1^ juice solution was added into each well. Reference antibiotic from Oxoid (Thermo Fisher Scientific IT, Milan, Italy) were used as specific positive controls: rifampicin (Rif) 30 µg/disk, ampicillin (Amp) 10 µg/disk, and oxacillin (Ox) 1 µg/disk. The Petri dishes were incubated at 37 °C for 24 h. After incubation, the diameter of the inhibition halo was measured. The experiment was performed in triplicate. Three readings were recorded, as recommended by the EUCAST (http://www.eucast.org) and [61] procedures.

#### 3.7.1. Broth Dilution Tests, MIC, and MBC

The minimum inhibitory concentration (MIC) and minimum bactericidal concentration (MBC) tests were performed according to EUCAST protocols, using the micro-broth dilution method with ½ serial dilutions in liquid medium [62,63]. Concentration ranging from 4 mg mL^−1^ to 0.039 mg mL^−1^ for each citrus juice were tested. Each strain was inoculated in the exponential (log) phase with a final concentration corresponding to 1 × 10^6^ CFU mL^−1^; the experiment was performed in triplicate and after 24 h of growth at 37 °C, the turbidity at λ = 620 nm of each set of combinations was measured. MIC was the lowest concentration that demonstrated bacteria growth inhibition, while MBC represented the lowest concentration able to reduce 99.9% of bacteria vitality (CFU mL^−1^), when the microbial suspensions was plated in agar medium.

#### 3.7.2. Anti-Biofilm Assay

Minimal biofilm inhibitory concentration (MBIC) for all citrus juices was evaluated following the modified crystal violet staining protocol described by Montana University Center for Biofilm Engineering (http://www.biofilm.montana.edu). In a multiwell plate containing 1 × 10^6^ CFU mL^−1^ of bacterial suspension, a serial concentration from 4 to 0.039 mg mL^−1^ of each citrus juice has been tested. Cultures were maintained at 37 °C for 3 days. After incubation, the plates were washed three times with PBS, then the biofilm adhering to well surface was stained with a 0.4% crystal-violet solution for 2 min. Following two washes with PBS, each well was added of 100 μl of 30% acetic acid. The biofilm was measured by a colorimetric assay at 620 nm by Multiskan FC Microplate Photometer (Thermo Fisher Scientific IT, Milan). MBIC was the juice concentration reporting an absorbance comparable with negative control (wells without bacteria).

### 3.8. Statistical Analysis

Statistical analysis was performed by GraphPad Prism 5 for Windows software (GraphPad Software, Inc., La Jolla, CA 92037, USA). Phenolics and ascorbic acid content of juices were expressed as mg L^−1^ of juice. Antioxidant activity was expressed as millimoles equivalents of ascorbic acid L^−1^ of juice. For analytical tests, a one-way ANOVA was performed to compare results obtained with different analytical methods, using a unifactorial complete randomized block design. Mean comparisons were calculated by Fisher’s least significant difference test at *p* ≤ 0.05.

Where not otherwise specified, biological tests were repeated four times. A one-way ANOVA was performed to highlight significant differences among treatments. The Student-Newman Keuls (SNK) test was used to separate the mean values (*p* ≤ 0.01). Mean values ± standard deviations (SD) are reported in figures.

For antimicrobial and antibiofilm assay, the evaluation of statistical significance of differences was performed using the Pearson’s chi-square test by statistical calculator (https://www.socscistatistics.com/tests/); a significant level of *p* < 0.05 was assumed.

## 4. Conclusions

In this paper some properties of Pompia juice were studied and compared with other commercially more widespread citrus species. Pompia juice was accurately characterized and new elements for a correct phylogeny were provided. The most represented flavonoids in the Pompia juice, are chrysoeriol-6,8-di-*C*-glucoside, isorhamnetin-7-rutinoside, diosmetin-6,8-dihexoside, and diosmin. Our characterization did not solve all existing doubts, but new analogies with lemon, citron, and bergamot have been found. Chemical analyzes highlighted the characteristics of a juice that is not drinkable as it is, but which instead possesses properties that suggest different uses. Analytical and electrochemical tests have shown a greater antioxidant capacity of Pompia than the other investigated species. It is mainly attributable to ascorbic acid and phenols with low redox potential with a specific significant contribution of isorhamnetin 3-*O*-rutinoside, a molecule with high reducing power, found in lemon in smaller quantities but not in orange juices. Biological assay demonstrated a lack of any apparent sign of systemic toxicity, and highlighted a strong effect against ROS and a significant capacity to protect cell membrane from ROS damages. Pompia juice, at very low concentration, also reduces the metabolic activity of colon cancer cell lines, although not as much as lemon, thus encouraging further insights. Finally, the results of tests on antimicrobial activity suggest that the analyzed juices have the ability to prevent the growth and formation of the biofilm of pathogenic microorganisms and that Pompia could be a raw material for obtaining natural antimicrobials.

Although further studies are needed for a complete characterization of the biological activities of Pompia juice, our data, together with the existing literature on the fruit, constitute a scientific basis for the promotion of the traditional utilization of Pompia, and suggest to make a wider use of this species in the food or pharmaceutical field.

## Figures and Tables

**Table 1:** Quantification (mg L^−1^ ± standard deviation) of phenolics in Pompia, lemon (*cv* Lisbon) and orange juices (*cv* Hamlin, Sanguinello and Moro) obtained by LCMS analysis.

Compounds	Phenolics Content (mg L^−1^ of Juice)				
Flavonoids	Pompia	Lemon	Orange		
			*(Hamlin)*	*(Sanguinello)*	*(Moro)*
Chrysoeriol 6,8-*C*-diglucoside (Stellarin-2)	109.2 ± 7.4	n.d.	n.d.	n.d.	n.d.
Apigenin 7-*O*-neohesperidoside (Rhoifolin)	n.d.	31.4 ± 11.1	n.d.	n.d.	n.d.
Diosmetin 6,8-diglucoside	54.5 ± 10.6	6.1 ± 0.6	n.d.	n.d.	n.d.
Apigenin 6,8-*C*-diglucoside (Vicenin 2)	n.d.	18.6 ± 1.0	61.8 ± 2.1	88.4 ± 2.9	41.4 ± 1.2
Naringenin 7-*O*-rutinoside (Narirutin)	n.d.	n.d.	100.1 ± 8.7	131.2 ± 14.8	12.8 ± 0.6
Isorhamnetin 3-*O*-rutinoside	79.4 ± 12.1	44.9 ± 6.7	n.d.	n.d.	n.d.
Apigenin 7-*O*-neohesperidoside 4′-glucoside (Rhoifolin 4-glucoside)	17.5 ± 2.1	< LOQ	n.d.	n.d.	n.d.
Eriodictyol 7-*O*-rutinoside (Eriocitrin)	11.5 ± 2.2	29.9 ± 4.1	< LOQ	< LOQ	< LOQ
Naringenin 7-*O*-neohesperidoside (Naringin)	n.d.	n.d.	132.6 ± 8.4	125.7 ± 6.6	39.3 ± 2.7
Hesperetin 7-*O*-rutinoside (Hesperidin)	7.1 ± 3.4	77.1 ± 6.2	422.8 ± 6.9	366.4 ± 18.6	182.3 ± 4.7
Diosmin	52.6 ± 8.8	25.7 ± 1.1	n.d.	n.d.	n.d.
Didymin	n.d.	n.d.	5.1 ± 0.2	16.2 ± 2.1	6.1 ± 0.2
Total	331.8	233.7	722.4	727.9	281.9
Anthocyanins					
Cyanidin 3-*O*-glucoside	n.d.	n.d.	n.d.	1.8 ± 0.4	118.9 ± 3.2
Cyanidin 3-*O*-(6″-malonyl-glucoside)	n.d.	n.d.	n.d.	1.0 ± 0.3	201.1 ± 4.9
Cyanidin 3-*O*-(6″-dioxalyl-glucoside)	n.d.	n.d.	n.d.	0.9 ± 0.3	31.7 ± 3.3
Total	n.d.	n.d.	n.d.	3.7	351.7
Total Phenolics	331.8	233.7	722.4	731.6	633.6

n.d. = not detected; LOQ = limit of quantification.

**Table 2:** (2A) Antioxidant capacity (ascorbic acid equivalents) as determined by SB electrochemical system at + 500 mV, by DPPH (EPR) and by ABTS (spectrophotometric) reference methods, in the juices of Pompia, lemon (*cv* Lisbon), and oranges (*cv* Hamlin, Sanguinello, and Moro). AA and phenolics contributions to antioxidant activity were also provided by SB system. (2B) Ascorbic acid content (mg L^−1^) as determined by SB system at + 500 mV, titrimetric, and HPLC methods in the juices of Pompia, lemon (*cv* Lisbon), and oranges (*cv* Hamlin, Sanguinello and Moro).

**(2A)–Antioxidant Capacity (mmoL Equivalents of AA L^−1^ of Juice)**									
			Citrus Species						
Method of Detection			Pompia	Lemon (Lisbon)		Orange (Hamlin)	Orange (Sanguinello)		Orange (Moro)
SB	Total		4.98 b (a)	4.59 b (a)		4.14 c (b)	3.89 b (c)		3.36 b (d)
	AA contribution		4.41 (a)	4.04 (b)		3.99 (b)	3.31 (c)		2.47 (d)
	Phenol contribution		0.57 (b)	0.55 (b)		0.15 (d)	0.58 (b)		0.89 (a)
DPPH	Total		5.16 b (a)	4.88 a (a)		4.46 b (b)	4.11 b (c)		3.88 a (c)
ABTS	Total		5.39 a (a)	5.09 a (ab)		4.83 a (b)	4.44 a (c)		4.12 a (c)

**(2B)–AA Content (mg L^−1^ of Juice)**									
		Method of Detection							
Citrus Species		SB			Titrimetric			HPLC	
Pompia		582.3 a (a)			545.2 a (b)			577.5 a (a)	
Lemon *cv* Lisbon		528.6 b (a)			496.0 b (a)			507.7 b (a)	
Orange *cv* Hamlin		582.0 a (a)			565 4 a (a)			574.4 a (a)	
Orange *cv* Sanguinello		503.7 b (a)			485.3 b (a)			493.2 b (a)	
Orange *cv* Moro		442.7 c (a)			n.d.			431.2 c (a)	

Means in columns followed by unlike letters differ significantly by Fisher’s least significant difference (LSD) procedure, *p* ≤ 0.05. Means in rows followed by (unlike letters) differ significantly by Fisher’s least significant difference (LSD) procedure, *p* ≤ 0.05. n.d.= not detectable.

**Table 3:** Antimicrobial profile for Pompia and lemon juices (4 mg mL^−1^) with a set of Gram-positive and Gram-negative bacteria.

Strain	Pompia	Lemon	**Control**
Gram negative			
*E. coli* DSM 1103	0.0 ± 0.0	0.0 ± 0.0	Amp. 19.6 ± 0.7
*P. aeruginosa* DSM 1117	9.1 ± 0.8	7.8 ± 1.4	Rif. 12.1 ± 0.5
*K. pneumoniae* DSM 681	0.0 ± 0.0	0.0 ± 0.0	Amp. 9.5 ± 0.8
Gram positive			
*S. aureus* DSM 1104	10.9 ± 1.1	10.2 ± 0.8	Ox. 22.6 ± 1.5
*E. faecalis DSM 2570*	11.2 ± 1.6	11.4 ± 2.2	Amp. 23.8 ± 0.9

Legend: Amp = ampicillin; Ox = oxacillin; Rif = rifampin.

**Table 4:** MIC, MBC, and MBIC values observed in selected Gram-positive and Gram-negative bacteria.

Strain	Lemon			Pompia		
	MIC	MBC	MBIC	MIC	MBC	MBIC
	mg mL^−1^			mg mL^−1^		
*P. aeruginosa* DSM 1117	4	>4	4	4	>4	2
*S. aureus DSM 1104*	2	>4	2	2	>4	1
*E. faecalis DSM 2570*	4	>4	4	4	>4	2

## Supplementary Materials

The following are available online: Figure S1: HPLC DAD chromatograms of Pompia, lemon Lisbon, and oranges Hamlin, Sanguinello and Moro juices; Table S1. Characterization of phenolics and organic compounds of Pompia, Lemon (*cv* Lisbon) and orange (*cv* Hamlin, Sanguinello and Moro) juice by LCMS analysis; Figure S2. Cyclic voltammetry in the absence and in the presence of 1 mM Isorhamnetin 3-O-rutinoside; Figure S3. Cyclic voltammetry in the absence and in the presence of 1 mM Diosmin; Figure S4. Cyclic voltammetry in the absence and in the presence of 1 mM Diosmetin 6,8-diglucoside; Figure S5. AA and quinic acid cyclic voltammetries; Figure S6. Cell viability of differentiated CaCo-2 cells treated with Pompia, Lemon (*cv* Lisbon) and orange (*cv* Hamlin, Sanguinello and Moro) juice at concentrations between 10 and 500 µg mL^−1^ in medium, and incubated for 72 h; Figure S7. Effect of Pompia and lemon juices on cells number of CaCo-2 and SW48 colon cancer cell lines.
