# Supplementary material for: Antioxidant, Antimicrobial, and Other Biological Properties of Pompia Juice
Source: Molecules. 2020 Jul 13;25(14):3186. doi: 10.3390/molecules25143186 (PMC7397052; doi:10.3390/molecules25143186)
Supplement: Supplementary file 1 [file molecules-25-03186-s001.pdf]

Suppplementary material

Antioxidant, Antimicrobial, and Other Biological Properties of Pompia Juice

Antonio Barberis [1,*,†], Monica Deiana [2,†], Ylenia Spissu [1,†], Emanuela Azara [3], Angela Fadda [1], Pier Andrea Serra [1,4], Guy D'hallewin [1], Marina Pisano [3], Gabriele Serreli [2], Germano Orrù [1,5], Alessandra Scano [5], Daniela Steri [6] and Enrico Sanjust [2,*]

[1] Institute of Sciences of Food Production, National Research Council, 07100 Sassari, Italy; yspissu82@gmail.com (Y.S.); angela.fadda@cnr.it (A.F.); paserra@uniss.it (P.A.S.); guy.dhallewin@cnr.it (G.D.); orru@unica.it (G.O.)

[2] Department of Biomedical Sciences, University of Cagliari, 09100 Cagliari, Italy; mdeiana@unica.it (M.D.); gabrieleserreli@hotmail.it (G.S.)

[3] Institute of Biomolecular Chemistry, National Research Council, 07100 Sassari, Italy; emanuelagigliola.azara@cnr.it (E.A.); marina.pisano@cnr.it (M.P.)

[4] Department of Medical, Surgical and Experimental Medicine, University of Sassari, 07100 Sassari, Italy

[5] Department of Surgical Sciences, Molecular Biology Service, University of Cagliari, 09100 Cagliari, Italy; alessandrascano@libero.it

[6] PPD Pharmaceutical Industries, 37121 Verona, Italy; daniela.steri@googlemail.com

* Correspondence: antonio.barberis@cnr.it (A.B.); sanjust@unica.it (E.S.); Tel.: +39-079-2841710 (A.B.)

† These authors contributed equally to this work.

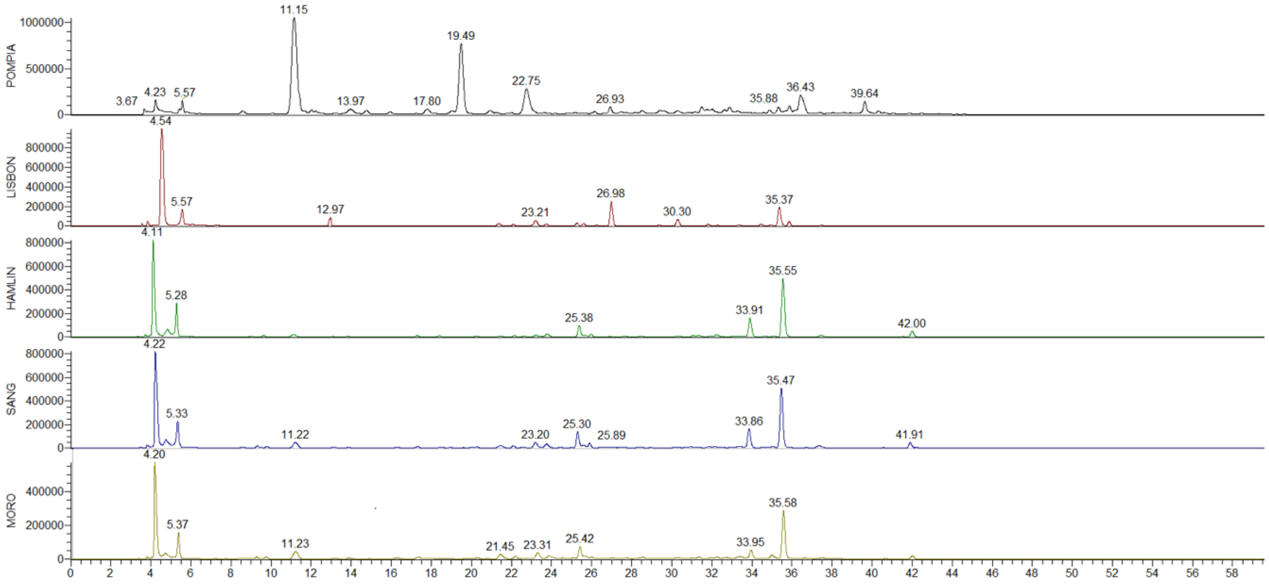

Figure S1. HPLC DAD chromatograms at 320 nm of Pompia, lemon Lisbon, and oranges Hamlin, Sanguinello and Moro juices.

Table S1. Characterization of phenolics and organic compounds of Pompia, Lemon (*cv* Lisbon) and orange (*cv* Hamlin, Sanguinello and Moro) juice by LCMS analysis.

Peak	RT (min)	Molecular formula	m/z calculate	m/z experimental	Δ (ppm)	Cultivar	Fragments	Compound
1	6.43	$C_6H_8O_7$	191.01973	191.01934	-2.04167	L	NEG	Citric acid
2	7.88	$C_9H_8O_3$	165.05462	165.05557	5.75567	P	POS	(E)-p-coumaric acid
3	11.15	$C_7H_{12}O_6$	191.05611	191.05699	4.60598	H,S,M,L,P	NEG	Quinic acid derivate
4	12.97	$C_{17}H_{20}O_9$	367.10342	367.0984	-0.01362	L	Pos	Feruloylquinic acid
5	17.74	$C_{21}H_{21}O_{11}$	450.11566	450.11285	-6.24284	M,S	POS	Cyanidin 3-*O*-glucoside
6	17.8	$C_{30}H_{36}O_{18}$	685.19744	685.1988	1.98483	P	POS	Caffeic acid hexoside dimer
7	19.49	$C_{28}H_{32}O_{16}$	625.17631	625.17284	-5.55043	P	POS	Chrysoeriol 6,8-*C*-diglucoside
8	20.94	$C_{28}H_{32}O_{16}$	625.17631	625.17284	-5.55043	P	POS	Diosmetin 6,8-*C*-dihexoside
9	21.67	$C_{27}H_{30}O_{14}$	577.15628	577.15453	-3.03211	L	NEG	Apigenin 7-*O*-neohesperidoside
10	22.75	$C_{28}H_{32}O_{15}$	609.18141	609.17875	-4.35010	P	POS	Diosmetin 7-*O*-neohesperidoside
11	22.8	$C_{28}H_{32}O_{16}$	625.17631	625.18009	6.04629	L,P	POS	Diosmetin 6,8 -*C*-diglucoside
12	22.9	$C_{25}H_{21}O_{17}$	594.08515	594.07993	-8.78662	M,S	POS	Cyanidin 3-6''-dioxalylglucoside
13	23.14	$C_{27}H_{30}O_{16}$	611.17631	611.17743	1.83253	L	POS	Luteolin 6,8-di-*C*-glucoside
14	23.21	$C_{33}H_{40}O_{21}$	773.21348	773.21492	1.86236	M,S,L,P	POS	Quercetin-7-O-glucoside 3-rutinoside
15	23.89	$C_{17}H_{22}O_{10}$	387.12857	387.13098	6.22532	H,M,S	POS	Sinapoyl D-glucoside
16	24.19	$C_{24}H_{23}O_{14}$	536.11606	536.11413	-3.59997	M,S	POS	Cyanidin-3-6'-malonylglucoside
17	25.18	$C_{27}H_{30}O_{15}$	595.16575	595.16181	-6.62000	H,M,S	POS	Apigenin 6,8-di-*C*-glucoside
18	25.22	$C_{27}H_{30}O_{15}$	593.15119	593.15211	1.55104	L	NEG	Luteolin 7-*O*-rutinoside
19	25.38	$C_{27}H_{32}O_{14}$	581.18648	581.18719	1.22164	H,M,S	POS	Naringenin 7-O-rutinoside
20	25.89	$C_{33}H_{42}O_{20}$	759.23422	759.22991	-5.67677	H,S,M	POS	Eriodictyol-7-O-rutinoside 4-glucoside
21	26.98	$C_{28}H_{32}O_{16}$	625.17631	625.17191	-7.03801	L,P	POS	Isorhamnetin-3-*O*-rutinoside
22	29.41	$C_{33}H_{40}O_{19}$	741.22366	741.22452	1.16024	L,P	POS	Rhoifolin 4-*O*-glucoside
23	30.3	$C_{27}H_{32}O_{15}$	597.18141	597.17923	-3.63374	H,S,M,L,P	POS	Eriodictyol 7-*O*-rutinoside
24	30.44	$C_{27}H_{30}O_{15}$	595.16575	595.17035	7.72894	H	POS	Kaempferol 3-*O*-rutinoside
25	31.35	$C_{28}H_{34}O_{15}$	611.19705	611.19297	-6.67542	H	POS	Hesperetin 7-O-neohesperidoside
26	31.82	$C_{27}H_{30}O_{16}$	611.16066	611.15873	-3.15793	H,L,P	POS	Luteolin-3',7-*O*-diglucoside
27	32.45	$C_{28}H_{32}O_{17}$	639.15667	639.15991	5.06918	M	NEG	Isorhamnetin 3',7-O--diglucoside
28	32.89	$C_{28}H_{34}O_{14}$	595.20213	595.19988	-3.78023	P	POS	Isosakuranetin 7-*O*-rutinoside
29	33.86	$C_{27}H_{32}O_{14}$	581.18648	581.18384	-4.54243	H,M,S	POS	Naringenin 7-*O*-neohesperidoside
30	33.91	$C_{22}H_{22}O_{11}$	463.1355	463.13495	-1.18756	M,S,L,P	POS	Diosmetin-7-*O*-glucoside
31	34.46	$C_{27}H_{30}O_{16}$	611.16066	611.16319	4.13966	L	POS	Quercetin 3-*O*-rutinoside
32	34.95	$C_{28}H_{32}O_{15}$	609.18104	609.17991	-1.85495	M,L,P	POS	Chrysoeriol 7-*O*-neohesperidoside
33	35.55	$C_{28}H_{34}O_{15}$	611.19705	611.19328	-6.16822	H,M,S,L,P	POS	Hesperetin 7-*O*-rutinoside
34	35.88	$C_{27}H_{32}O_{14}$	581.18648	581.18712	1.10120	P	POS	Naringenin 5-*O*-neohesperidoside
35	36.43	$C_{28}H_{32}O_{15}$	609.18104	609.17727	-6.18864	L,P	POS	Diosmin
36	36.88	$C_{26}H_{30}O_8$	471.20134	471.20387	5.36925	L	POS	Limonin
37	37.48	$C_{29}H3_4O_{17}$	653.17232	653.17272	0.61240	L	NEG	Neo-Limocitrin
38	38.94	$C_{33}H_{40}$O20	757.21857	757.21299	6.49545	P	POS	Kaempferol 3-*O*-rutinoside 7-O glucoside
39	39.64	$C_{33}H_{40}O_{20}$	757.21857	757.22211	4.67500	P	POS	Quercetin 3-O-rhamnosyl-rhamnosyl-glucoside
40	42.01	$C_{28}H_{34}O_{14}$	595.20213	595.20018	-3.27620	H,M,S	POS	Dydimin
41	44.37	$C_{28}H_{34}O_9$	515.22757	515.22482	-5.33745	L	POS	Nomilin
42	45.02	$C_{16}H_{12}O_7$	317.06558	317.06377	-5.70860	L	POS	Isorhamnetin
43	50.36	$C_{15}H_{10}O_8$	319.04484	319.04516	1.00299	P	POS	Myricetin
44	51.74	$C_{15}H_{10}O_7$	303.04993	303.04879	-3.76176	H	POS	Quercetin
45	53.55	$C_{20}H_{20}O_7$	373.12818	373.13035	5.81570	H	POS	Sinensetin
46	55.77	$C_{21}H_{22}O_8$	403.13874	403.14051	4.39055	H	POS	Nobiletin

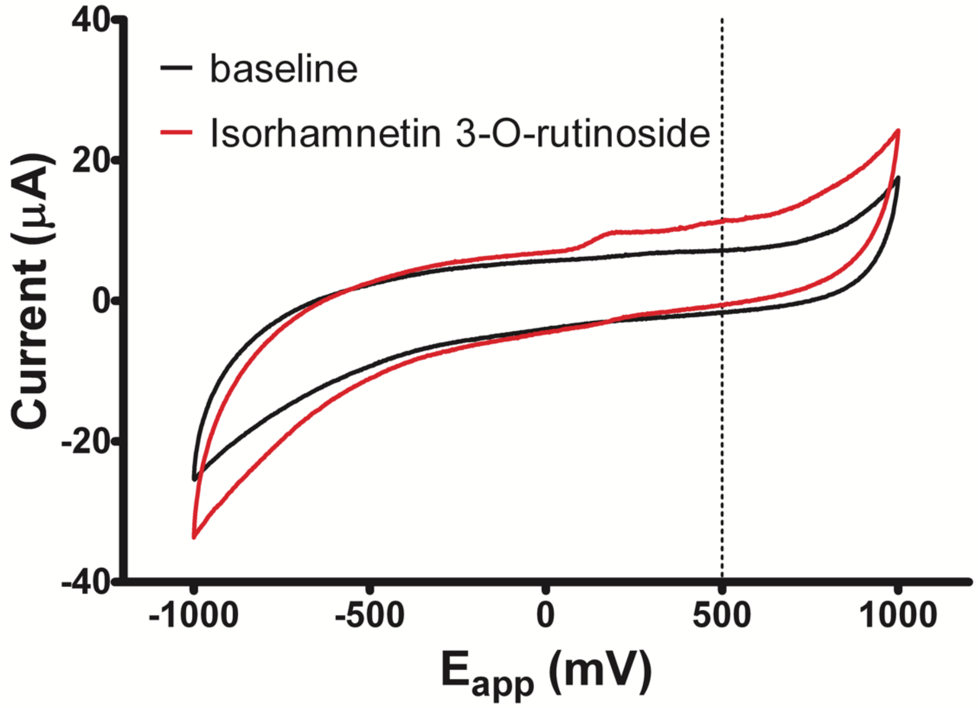

Figure S2. Cyclic voltammetry, with a scanned potential range (E_{app}) comprised between – 1 V and + 1 V *vs* carbon pseudoreference, in the absence (PBS black line) and in the presence of 1 mM Isorhamnetin 3-O-rutinoside (red line).

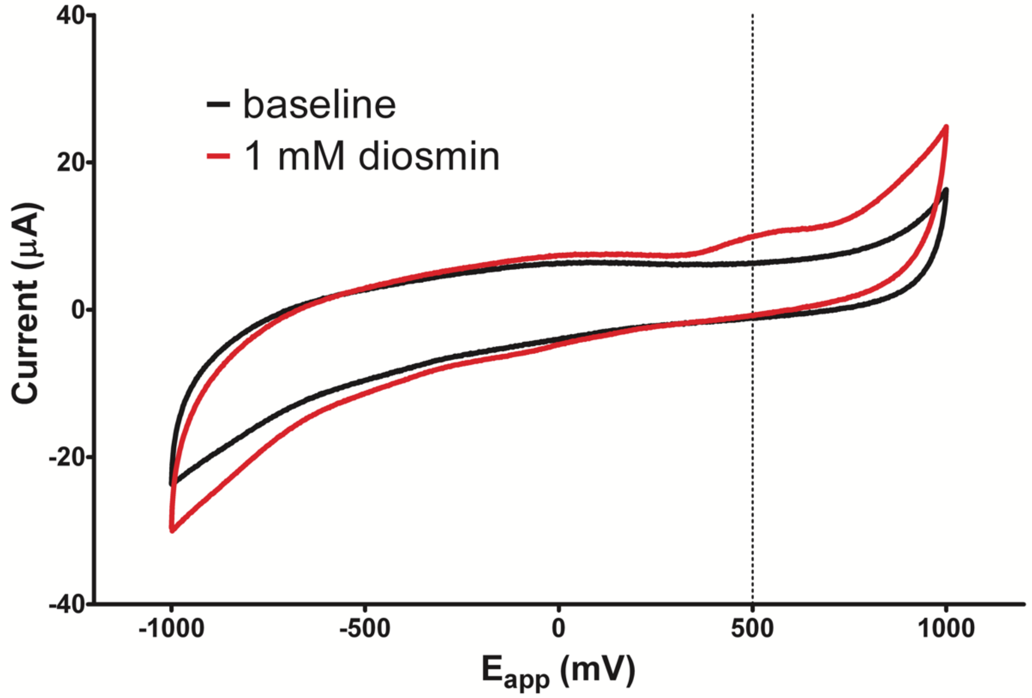

Figure S3. Cyclic voltammetry, with a scanned potential range (E_{app}) comprised between – 1 V and + 1 V *vs* carbon pseudoreference, in the absence (PBS black line) and in the presence of 1 mM Diosmin (red line).

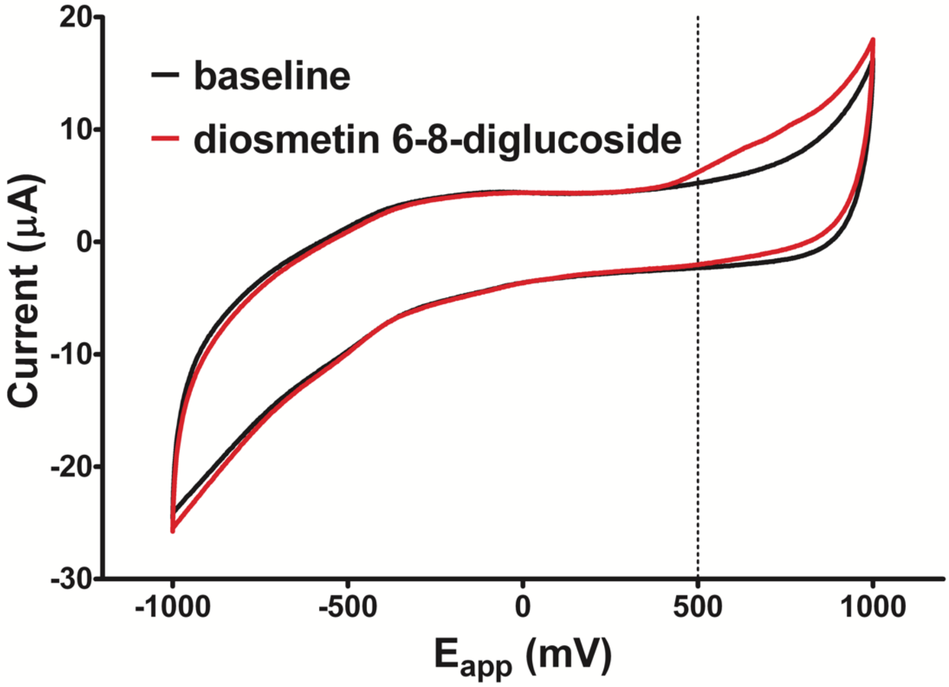

Figure S4. Cyclic voltammetry, with a scanned potential range (E_{app}) comprised between – 1 V and + 1 V *vs* carbon pseudoreference, in the absence (PBS black line) and in the presence of 1 mM Diosmetin 6,8-diglucoside (red line).

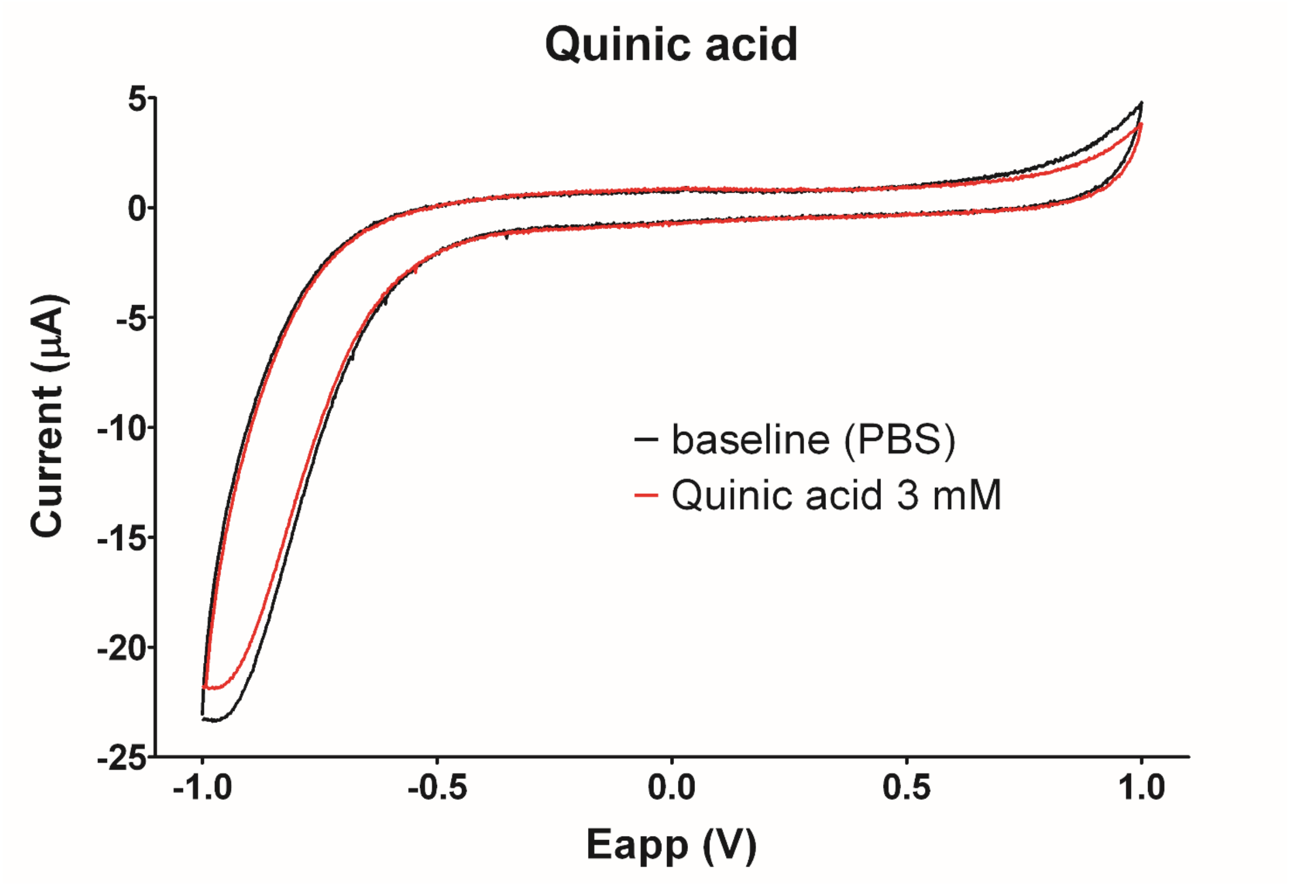

Figure S5. AA and quinic acid cyclic voltammetries, with a scanned potential range (E_{app}) comprised between – 1 V and + 1 V *v*s carbon pseudoreference, in the absence (PBS black line) and in the presence of 3 mM quinic acid (red line).

MTT assay on Caco-2 cells

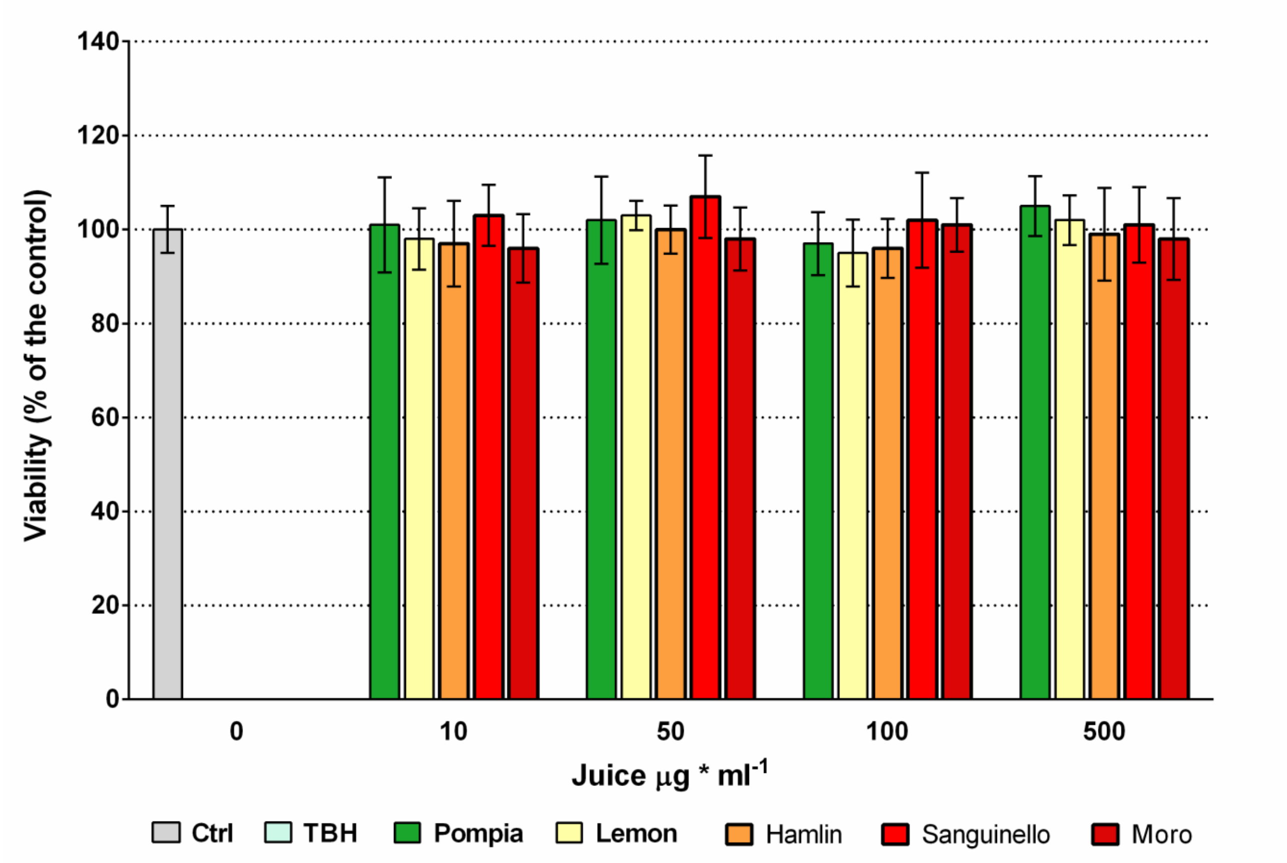

Figure S6. Cell viability of differentiated CaCo-2 cells treated with Pompia, Lemon (*cv* Lisbon) and orange (*cv* Hamlin, Sanguinello and Moro) juice at concentrations between 10 and 500 µg * mL^{-1} in medium, and incubated for 72 h. Data are presented as mean ± SD (n = 4). * = $p \leq 0.01$ *vs* control.

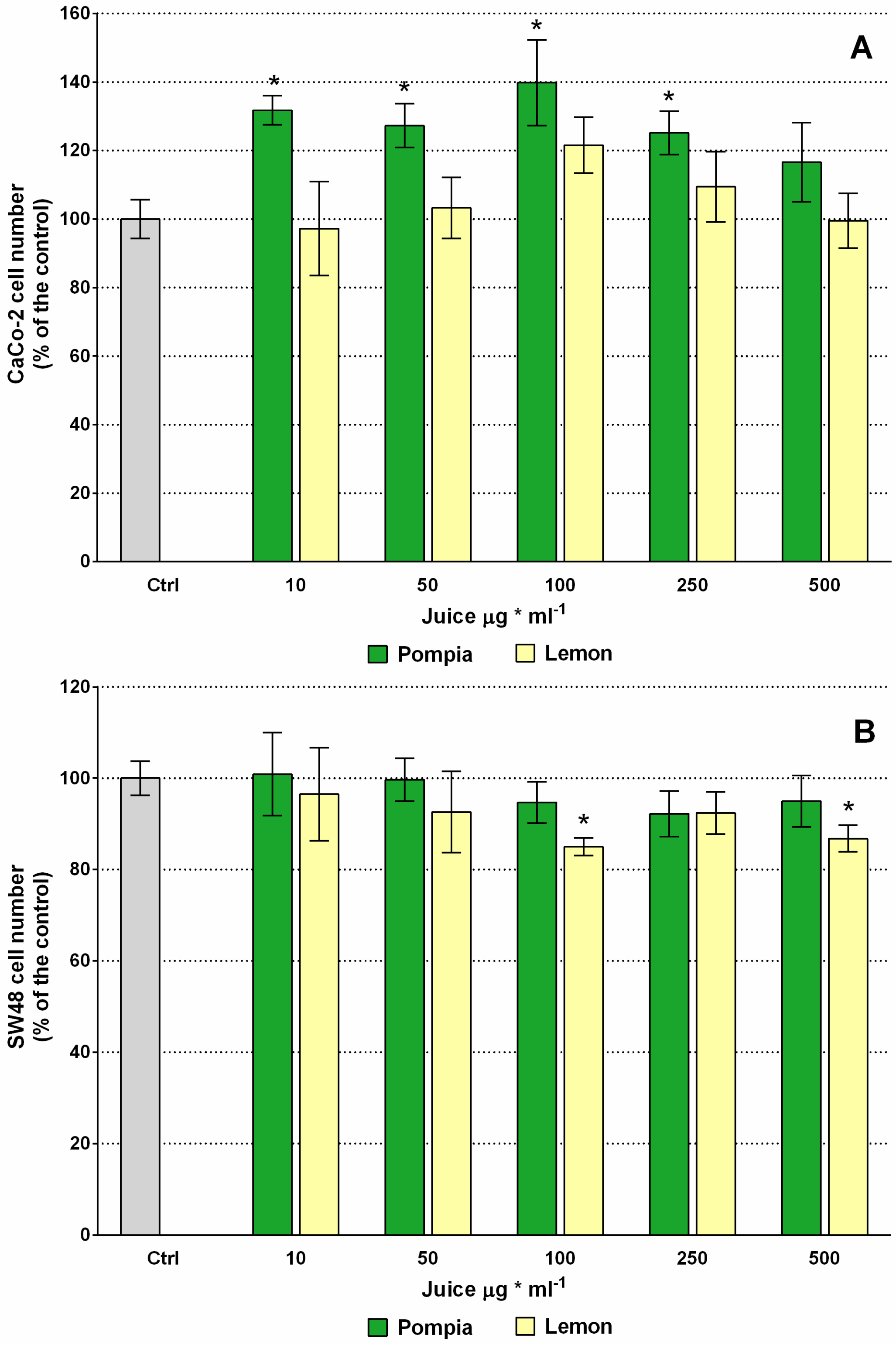

Figure S7. Effect of Pompia and lemon juices on cells number of CaCo-2 (A) and SW48 (B) colon cancer cell lines.
